# The "Women's Ambulance" in a Shell Factory

**Published:** 1918-08-24

**Authors:** 


### THE "WOMEN'S AMBULANCE" IN A SHELL FACTORY.

A SHELL-FACTORY NURSE sends us the following account of the nursing service for minor injuries which is run as part of the Welfare Scheme in a shell factory :

This service has been in operation for eighteen months —long enough to prove its worth and usefulness. It provides dressings for minor injuries, first aid for serious accidents, and treatment in sudden illness, also simple remedies for headache, indigestion, and slight illness, and in so doing prevents infection of slight wounds and the necessity of going home with a headache or other temporary illness, as provision is made for resting for an hour or two. It also gives a sense of security to the girls and overseers, especially on the "night shift." The days vary, sometimes just cuts and headaches and indigestion. Then a sudden commotion, and someone says "Nurse! an accident," and a girl is carried or helped in, probably with a crushed hand or foot.

They are very brave, these girls, and seldom say a word while the hand is "cleaned up," painted with tincture of iodine, and made as comfortable as possible. A motor is brought, and there is a nineteen-mile run to the city and the infirmary. There are two nurses on each shift—a charge nurse and an assistant. The charge nurses are fully qualified, with maternity experience, and the assistants, at present, fever-hospital trained.

The work is part of the Welfare Scheme, and has amply justified itself, as we have better health, better time-keeping, and better output than before, in spite of the fact that we are getting a poorer class of girls, "war weariness" worry, and, not least, the food question. We have not the advantage of new buildings. The factory was built for a different purpose, and is not convenient. The girls are scattered over several shops, so we have two rooms, one in each end as it were. We dream of a "Central Ambulance," but that is not yet realised. It is a "two-shift" factory, and that means long hours—eleven hours on day shift and twelve on night shift.

The cost of the Service, not including housing and equipment, was last year about £5 per month for an average of 1,400 to 1,500 girls. The salaries of the nurses and assistants were £35 per month, making a total running expense of £40 per month for 1,500 girls. This sum is a fraction less than 6½d. per month per employee, a small cost surely for all the security and benefit derived. In a recent article on the "Industrial Nurse" this statement is made: "The salary is not good." Of course nurses' salaries in Great Britain are "not good," but I think ours compare favourably with most branches of nursing. I started with £90 and a £20 bonus for "night shift." At the end of a year's service an advance of £10, and now, as from October, in common with the other women on the factory staff, another increase—"to meet the increased cost of living"—brings my salary, including bonuses, to almost £160 per annum.

The hours are long, but the weeks are not—five and a-half days on day shift and, at present, five nights on night shift, and long may the Sunday night shift be "off"! This gives us always the week-ends free. Only those who for years have had seven working days a week fully appreciate this arrangement During the year I have worked several Sunday nights and on holidays and Saturdays. On the other hand, I have had some time off to make up for it.

I can furnish statistics and a detailed account of necessary equipment, treatment used, and results obtained if it would be of interest.

